# Role of Cytochrome P450 Enzymes in the Metabolic Activation of Tyrosine Kinase Inhibitors

**DOI:** 10.3390/ijms19082367

**Published:** 2018-08-11

**Authors:** Klarissa D. Jackson, Rebecca Durandis, Matthew J. Vergne

**Affiliations:** College of Pharmacy and Health Sciences, Lipscomb University, Nashville, TN 37204, USA; rdurandis@mail.lipscomb.edu (R.D.); matt.vergne@lipscomb.edu (M.J.V.)

**Keywords:** tyrosine kinase inhibitor, bioactivation, cytochrome P450, hepatotoxicity

## Abstract

Tyrosine kinase inhibitors are a rapidly expanding class of molecular targeted therapies for the treatment of various types of cancer and other diseases. An increasing number of clinically important small molecule tyrosine kinase inhibitors have been shown to undergo cytochrome P450-mediated bioactivation to form chemically reactive, potentially toxic products. Metabolic activation of tyrosine kinase inhibitors is proposed to contribute to the development of serious adverse reactions, including idiosyncratic hepatotoxicity. This article will review recent findings and ongoing studies to elucidate the link between drug metabolism and tyrosine kinase inhibitor-associated hepatotoxicity.

## 1. Introduction

Improved understanding of the signaling pathways that regulate cellular functions, including growth and proliferation, has led to the identification of kinases as key drug targets [1]. Dysregulation of receptor and non-receptor tyrosine kinases is a common feature in tumor development and cancer progression [2]. Thus, pharmacological inhibition of tyrosine kinases has been established as a clinically useful approach for the treatment of various types of cancer, and other diseases [2].

Small molecule kinase inhibitors are a rapidly growing class of targeted therapies, with the largest impact in cancer treatment. Imatinib was the first successful small molecule tyrosine kinase inhibitor approved by the US Food and Drug Administration (FDA) and the European Medicines Agency (EMA) in 2001 for the treatment of chronic myeloid leukemia [3]. Since that time, over 30 small molecule kinase inhibitors have been approved for clinical use in cancer therapy and other diseases (Table 1) [4]. Tyrosine kinase inhibitors represent approximately 75% of all small molecule kinase inhibitors [4]. The therapeutic indications for tyrosine kinase inhibitors include the treatment of several types of cancer, such as leukemia, lung, breast, kidney, gastrointestinal, and skin cancers. A smaller number of kinase inhibitors have non-cancer indications, including rheumatoid arthritis (tofacitinib) and pulmonary fibrosis (nintedanib) [4]. Several other kinase inhibitors are currently in clinical trials. 

One of the assumed benefits of molecular targeted anticancer agents is that they generally lack the cytotoxic effects of traditional chemotherapies; however, they introduce new challenges to optimize drug therapy [5]. Unlike traditional chemotherapies, small molecule tyrosine kinase inhibitors are administered orally, and most are administered at fixed doses. Severe toxicities, including hepatotoxicity and cardiotoxicity, limit the use of tyrosine kinase inhibitors in some patients. 

Drug-induced liver injury (hepatotoxicity) has been associated with several tyrosine kinase inhibitors in clinical use [6,7,8,9]. Mandatory black-box warnings for severe and fatal idiosyncratic hepatotoxicity have been issued for five tyrosine kinase inhibitors: lapatinib, sunitinib, pazopanib, regorafenib, and ponatinib. The non-tyrosine kinase inhibitor idelalisib also carries a black-box warning for hepatotoxicity. The underlying mechanisms of hepatotoxicity associated with these agents remain largely unknown. This represents a significant challenge in cancer therapy and drug development. Understanding the mechanisms and risk factors of drug-induced liver injury has major implications for improving prediction and prevention of these events [10].

Metabolic activation (bioactivation) of small molecule kinase inhibitors by cytochrome P450 (CYP) enzymes leading to formation of chemically reactive products is proposed as a key initiating event in tyrosine kinase inhibitor-induced hepatotoxicity. Several tyrosine kinase inhibitors have been shown to undergo bioactivation to form reactive metabolites. This topic has been reviewed, with excellent articles by Duckett and Cameron, [11] Stepan et al. [13] and Teo et al. [14] Reactive metabolites have been identified for the following clinically available tyrosine kinase inhibitors: dasitinib [15], gefitinib [16]. erlotinib [17], lapatinib [18,19], imatinib [20], axitinib [21], ponatinib [22], sunitinib [23], as well as investigational tyrosine kinase inhibitors. The purpose of the present review is to (1) provide updates on recently characterized bioactivation mechanisms of selected tyrosine kinase inhibitors, (2) discuss progress towards elucidating the cellular mechanisms of hepatocellular injury related to tyrosine kinase inhibitors, and (3) briefly discuss recent findings related to risk factors of tyrosine kinase inhibitor-induced hepatotoxicity. 

## 2. Bioactivation of Small Molecule Tyrosine Kinase Inhibitors

Screening approaches to detect the formation of reactive electrophilic metabolites have been well established. These approaches often involve the use of trapping agents, such as glutathione (GSH), potassium cyanide (KCN), and methoxylamine in incubations with human liver microsomes or S9 fraction (cytosol + microsomes) fortified with nicotinamide adenine dinucleotide phosphate (NADPH) [24,25,26,27]. It should also be noted that the mechanism of action of four of the current FDA-approved tyrosine kinase inhibitors involves covalent modification and irreversible inhibition of their pharmacologic targets. These drugs and their targets include ibrutinib (BTK), afatinib (EGFR, HER2, HER4), osimertinib (EGFR), and neratinib (EGFR, HER2, HER4). 

### 2.1. Screening for Time-Dependent Inhibition and Reactive Metabolite Formation 

Metabolism by CYP3A enzymes is the predominate route of drug elimination for most small molecule tyrosine kinase inhibitors [11,28]. CYP3A enzymes have been shown to play a major role in drug bioactivation; CYP1A enzymes are also reported to catalyze the bioactivation of some tyrosine kinase inhibitors. Recent studies indicate that many tyrosine kinase inhibitors cause time-dependent inhibition of cytochrome P450 enzymes, particularly CYP3A, in vitro [26]. Mechanism based-inactivation of CYP3A has been characterized for dasatinib [15], lapatinib [18,29], axitinib [21], lestaurtinib, and saracatinib [30]. 

The following studies utilized P450 inactivation parameters to assess the potential for drug–drug interactions with various kinase inhibitors. Kenny et al., reported a systematic screen of tyrosine kinase inhibitors to evaluate time-dependent P450 inhibition and assess the formation of reactive metabolites [26]. This analysis included nine tyrosine kinase inhibitors, eight of which were found to cause time-dependent inhibition (TDI) of cytochrome P450 enzymes—most commonly CYP3A [26]. The TDI was determined by the shift in the area under the curve (AUC) of the IC_50_ curve. Testosterone and midazolam were both used as probe substrates for CYP3A. Detailed kinetic parameters (K_I_, k_inact_) were also determined for TDI-positive compounds. Evidence of reactive metabolite formation was found for nine of the tyrosine kinase inhibitors tested (dasatinib, erlotinib, gefitinib, imatinib, lapatinib, nilotinib, pazopanib, sorafenib, and sunitinib) [26]. Tyrosine kinase inhibitors were listed with the intensity and number of GSH, cyanide, and methoxylamine conjugates formed [26].

In another study, Filppula et al. screened 14 kinase inhibitors for time-dependent inhibition of CYP3A and CYP2C8 [30]. Amodiaquine *N*-deethylation was used as the marker reaction for CYP2C8 activity, and midazolam 1’-hydroxylation was used as the marker for CYP3A activity in human liver microsomes [30]. Time-dependent inhibition was assessed by IC_50_ shift assay with the 14 kinase inhibitors. Detailed mechanism-based inactivation was characterized for bosutinib, lestaurtinib, and saracatinib. Erlotinib and gefitinib were found to activate/stimulate midazolam 1′-hydroxylation in experiments without pre-incubation [30]. Bosutinib was found to cause time-dependent inhibition of CYP2C8-mediated amodiaquine *N*-deethylation (“weak mechanism-based inactivation”), and lestaurtinib and saracatinib were found to cause time-dependent inhibition (mechanism-based inactivation) of CYP3A-mediated midazolam 1′-hydroxylation [30]. 

Wang et al. also assessed the ability of 12 small molecule kinase inhibitors to inhibit CYP2C8- and CYP3A4-mediated metabolism of paclitaxel, a cytotoxic agent commonly used in combination cancer therapy [21]. In addition to kinase inhibitors previously shown to inactivate CYP3A4, axitinib was also found to inactivate CYP3A4 in a time- and concentration-dependent manner.

### 2.2. Bioactivation Mechanisms of Specific Tyrosine Kinase Inhibitors

The bioactivation mechanisms of several tyrosine kinase inhibitors have been well defined. Previous reviews [11,13,14] have focused on the bioactivation pathways of dasitinib [15], gefitinib [16], erlotinib [17], and lapatinib [18] (Table 2). Briefly, dasatinib was shown to undergo bioactivation by CYP3A4 to form quinoneimine and imine methide intermediates [15]. CYP3A-mediated bioactivation of gefitinib [16], erlotinib [17], and lapatinib [18] results in the formation of quinoneimine intermediates. Extrahepatic CYP1A1 was also shown to contribute to metabolic activation of gefitinib and erlotinib [16,17]. Reactive metabolites of these drugs were trapped as stable GSH conjugates. More recently, Zhao et al. described a novel bioactivation pathway of erlotinib mediated by CYP3A4 and CYP3A5 to form a reactive ketene intermediate, which was trapped as an adduct with 4-bromobenzylamine [31]. In addition to these tyrosine kinase inhibitors, imatinib [20], axitinib [21], ponatinib [22], and sunitinib [23], have been shown to undergo bioactivation to form reactive metabolites, which were trapped as stable adducts. Recent findings regarding the bioactivation mechanisms of these tyrosine kinase inhibitors will be discussed below.

#### 2.2.1. Imatinib

Imatinib, an inhibitor of breakpoint cluster region protein (BCR)-ABL, platelet-derived growth factor receptor (PDGFR), and c-Kit, was the first FDA-approved small molecule tyrosine kinase inhibitor, indicated for the treatment of Philadelphia chromosome-positive (Ph^+^) chronic myeloid leukemia (CML), acute lymphatic leukemia (ALL), and other types of cancer [3]. In the study by Kenny et al., seven cyanide conjugates and one methoxylamine conjugate were observed from liver microsomal incubations in vitro [26]. Li et al., characterized the bioactivation of imatinib at the piperizine ring to form imine and imine–carbonyl (α,β-unsaturated) intermediates, and bioactivation of the *p*-toluidine moiety to form imine–methide intermediates trapped as cyanide adducts [20].

#### 2.2.2. Axitinib

Axitinib is an inhibitor of VEGFR approved for the treatment of advanced renal cell carcinoma [32]. As described above, axitinib was found to cause TDI of CYP3A4 [21]. In studies to examine the mechanism of CYP3A4 inactivation by axitinib, two GSH conjugates of axitinib were detected [21]. One GSH conjugate was formed in the absence of NADPH, and the other GSH conjugate was formed in an NADPH-dependent manner, suggesting P450-mediated bioactivation for the later. The bioactivation mechanism of axitinib was proposed to involve epoxidation [21]. 

#### 2.2.3. Ponatinib

Ponatinib is an inhibitor of BCR-ABL tyrosine kinase and is approved for the treatment of resistant CML and Philadelphia chromosome-positive ALL [35]. Lin et al., studied ponatinib bioactivation in vitro, and ponatinib pharmacokinetics in vivo, in wild-type and humanized CYP1A1/2 mice [22]. In incubations of ponatinib with recombinant P450 enzymes supplemented with NADPH and GSH, GSH conjugates of ponatinib were formed primarily by CYP1A1 [22]. Co-incubation with recombinant mouse glutathione *S*-transferase (mGstp 1) enhanced the formation of GSH conjugates. Ponatinib–Gstp1 adducts were also detected from incubations of ponatinib with CYP1A1 and Gstp1 [22]. The proposed ponatinib bioactivation pathway was suggested to involve epoxide formation [22] however, the detailed reaction mechanism has not been characterized. 

In vivo, wild-type mice were pretreated with the aryl hydrocarbon receptor (AhR) activator TCDD (2,3,7,8-tetrachlorodibenzodioxin), and humanized CYP1A1/2 mice were pretreated with 3-methylcholanthrene (3-MC) followed by administration of ponatinib. Activation of AhR in wild-type mice resulted in increased clearance of ponatinib leading to 87% reduction in AUC and 62% decrease in half-life compared to untreated wild-type mice [22]. Ponatinib clearance was also increased in humanized CYP1A1/2 mice pretreated with 3-MC (54% reduction in AUC and 67% reduction in half-life) compared to untreated humanized mice. Ponatinib GSH conjugates were detected in fecal samples of 3-MC pretreated humanized CYP1A1/2 mice, but not in the feces of vehicle-treated mice [22]. The results of this study indicate that CYP1A induction may enhance formation of ponatinib reactive metabolites in vivo [22]. Since CYP1A1 is not appreciably expressed in liver, the role of CYP1A1 in ponatinib bioactivation may be important in extra-hepatic tissues, such as lung [36].

#### 2.2.4. Sunitinib

Sunitinib is an inhibitor of multiple tyrosine kinases, including VEGFR, PDGFR, and c-Kit, and is approved for the treatment of metastatic renal cell carcinoma, imatinib-resistant gastrointestinal stromal tumors, and pancreatic neuroendocrine tumors [37,38]. As stated above, Kenny et al., reported the formation of reactive metabolites from sunitinib in vitro based on detection of two GSH conjugates; however, the structures of the reactive metabolites were not characterized [26]. Later, Xie et al. demonstrated formation of reactive metabolite–GSH conjugates from sunitinib and its structural analog famitinib in human microsomal incubations [39]. These findings prompted further investigations into the bioactivation pathways of sunitinib. Amaya et al., recently reported identification of the cytochrome P450 enzymes involved in sunitinib metabolic activation [23]. A putative quinoneimine reactive metabolite trapped as a GSH conjugate was detected from human liver microsomal incubations supplemented with NADPH and GSH. CYP1A2 and CYP3A4 were shown to be the primary hepatic enzymes involved in sunitinib bioactivation, and CYP1A1, an extrahepatic P450, also generated reactive metabolite–GSH conjugates. The bioactivation mechanism was proposed to involve oxidative defluorination and quinoneimine formation, which has been previously described for famitinib and gefitinib [16,39]. Notably, formation of GSH conjugates of the putative sunitinib quinoneimine was higher in human liver microsomes with high CYP1A2 activity compared to human liver microsomes with low CYP1A2 activity [23]. Since smoking is known to induce CYP1A enzymes, it is possible that smoking may increase the generation of sunitinib reactive metabolites. The effect of smoking on sunitinib metabolism in vivo warrants further investigation.

The parent drug sunitinib contains a chemically reactive α,β-unsaturated carbonyl moiety, which can undergo Michael addition with cellular thiols, such as GSH and cysteine residues of proteins. Thus, GSH conjugates can form directly from the parent drug. GSH conjugates of sunitinib parent drug were detected in incubations without NADPH, which demonstrates the intrinsic reactivity of the drug [23]. Additional studies are required to determine whether the parent drug sunitinib and/or its reactive quinoneimine metabolite exert organ toxicities due to covalent binding of cellular proteins, oxidative stress, or other mechanism(s). 

### 2.3. Bioactivation of Investigational Tyrosine Kinase Inhibitors

#### 2.3.1. Saracatinib

Saracatinib (AZD-0530) is an inhibitor of the Src kinase family and BCR-ABL tyrosine kinase, and this compound is in clinical trials for anticancer therapy [40]. Saracatinib contains a 1,3-benzodioxole moiety and an *N*-methyl piperazine group. Chen et al., demonstrated that saracatinib undergoes bioactivation predominately by CYP3A4 [33]. The proposed bioactivation mechanism involved demethylenation of the 1,3-benzodioxole moiety to form a catechol and further oxidation to generate an electrophilic ortho-quinone, which was trapped as a GSH conjugate [33]. The saracatinib GSH conjugate was observed in vitro in human and rat microsomal incubations, and in vivo in the bile of rats treated with saracatinib [33]. This bioactivation pathway is suggested to contribute to inactivation of CYP3A and saracatinib-induced toxicities, including pulmonary and hepatic toxicity [33].

Saracatinib was further characterized as a mechanism-based inactivator of CYP3A [30]. In time-dependent inhibition IC_50_ shift assays with saracatinib, the IC_50_ for CYP3A shifted from IC_50_ = 46.0 μM with no preincubation to 1.8 μM with a 30 min preincubation, representing a 26-fold reduction in IC_50_ [30]. The inhibition occurred in a time-, inhibitor concentration-, and NADPH-dependent manner. The inactivation parameters of CYP3A by saracatinib were *K_I_* = 12.6 μM, and *k*_inact_ = 0.096 min^−1^ [30]. Thus, CYP3A inactivation by saracatinib could occur at therapeutic concentrations. Saracatinib was predicted to cause a ≥2.7-fold increase in the AUC of CYP3A substrates [30]. In addition to GSH conjugates, Attwa et al., recently reported detection of three cyano adducts derived from iminium intermediates from bioactivation of the piperizine ring of saracatinib in rat human liver microsomes [41].

#### 2.3.2. Masitinib 

Masitinib is a tyrosine kinase inhibitor that has anti-inflammatory and antitumor activity. In vitro, masitinib has an inhibitory effect on c-Kit wild type and mutate forms (exon 9 and 11) [42]. Masitinib is currently in clinical trials for the treatment for Alzheimer’s disease, gastrointestinal stromal tumors, and amyotrophic lateral sclerosis. Masitinib is structurally similar to imatinib, a tyrosine kinase inhibitor for which multiple cases of liver injury have been reported [27]. A European Medicines Agency assessment report for masitinib reported that the major metabolite of masitinib in mice, rats, dogs, and humans is the product of masitinib demethylation [43]. A genotoxic metabolite of masitinib was also reported. In a study by Amer et al., multiple metabolites of masitinib were identified from incubations with rat liver microsomes [34]. Eight cyano adducts were detected from microsomal incubations with potassium cyanide. The bioactivation reactions were proposed to occur on the piperazine moiety of masitinib to generate imine and imine–carbonyl intermediates, similar to the bioactivation of imatinib [34].

## 3. Downstream Toxicity Mechanisms of Tyrosine Kinase Inhibitors

Several mechanisms have been proposed to contribute to drug-induced liver injury, and it is likely that multiple concerted pathways are involved [44,45,46]. Formation of reactive metabolites is hypothesized to be a key initiating event in tyrosine kinase inhibitor-induced hepatotoxicity. Reactive electrophilic metabolites can covalently modify cellular proteins and/or induce reactive oxygen species production (oxidative stress), potentially leading to direct cell stress, mitochondrial dysfunction, and/or activation of immune-mediated reactions [10,47]. Alternatively, disruption of bile acid homeostasis due to inhibition of hepatobiliary transporters by drugs or drug metabolites has been shown to play a role in cholestatic liver injury [45,48,49]. 

Few studies have examined the downstream cellular effects of tyrosine kinase inhibitors and their metabolites as it relates to the pathogenesis of drug-induced liver injury. The mechanisms remain largely unknown; however, recent studies have begun to provide insight into the cellular pathways of tyrosine kinase inhibitor-induced hepatotoxicity. Much of the work in these studies has focused on the effects of tyrosine kinase inhibitors on mitochondrial function and cell signaling pathways leading to toxicity. 

Finding the appropriate cell model for detailed mechanistic studies on drug-induced liver injury is a challenge. A key step in elucidating the underlying mechanisms of drug-induced hepatotoxicity is to determine whether the toxicity arises from the parent drug or its metabolite(s). Use of a metabolically competent hepatic cell model is preferred; however, the availability of primary hepatocytes, reproducibility, and technical ease of use can be limitations. The metabolically competent human HepaRG cell line is a useful tool because, among other features, CYP expression is inducible using prototypical inducers [50,51,52,53,54]. Many mechanistic toxicologic studies are performed using the hepatoma cell line HepG2 cells. A key limitation to this model is that HepG2 cells do not have significant cytochrome P450 drug metabolism activity. Various approaches can be employed to optimize this common in vitro system, such as the use of HepG2 cells transfected with CYP enzymes. Biochemical manipulations in HepG2 may also be useful in addressing specific mechanistic questions. For example, depriving cells of glucose and supplementing growth media with galactose requires cells to utilize the mitochondrial respiratory chain to generate ATP instead of relying on glycolysis for ATP, and this sensitizes cells to mitochondrial toxicants [55]. Culturing cells under glucose conditions favors glycolysis, and culturing cells under galactose conditions favors mitochondrial metabolism. Thus, comparing the viability of cells grown in glucose vs galactose is used experimentally to detect drugs that cause mitochondrial dysfunction [55]. 

The nuclear factor erythroid 2-related factor (Nrf2) pathway is activated in response to oxidative and electrophilic stress, leading to transcription of cytoprotective genes, such as NAD(P)H: quinone oxidoreductase 1 (NQO-1) and heme-oxygenase 1 (HO-1). Cellular electrophilic stress is sensed by Keap 1 in the cytosol [56,57]. In the presence of oxidative and electrophilic stress, Nrf2 translocates from the cytosol to the nucleus, where it binds with its heterodimer partner musculo-aponeurotic factor protein (Maf) to the antioxidant response element (ARE) to activate gene transcription. Thus, activation of the antioxidant response via the Nrf2 pathway may also be monitored as an indicator of drug-induced cell stress. The use of these and other approaches to study tyrosine kinase inhibitor-associated hepatotoxicity is discussed below. 

### 3.1. Dasatinib

Dasatinib, an inhibitor of BCR-ABL and SRC kinases, is a second-generation tyrosine kinase inhibitor approved for the treatment of imatinib-resistant chronic myeloid leukemia (CML) and Philadelphia chromosome-positive acute lymphoblastic leukemia (ALL). Xue et al., examined dasatinib-induced hepatic injury in rat primary hepatocytes and in vivo in rats treated with dasatinib [58]. Dasatinib induced hepatic injury in vitro in a time- and dose-dependent manner in rat primary hepatocytes [58]. Treatment of rats with dasatinib (25 mg/kg/day) for 10 days increased liver injury markers, including elevated serum alanine aminotransferase (ALT), aspartate aminotransferase (AST), and lactate dehydrogenase (LDH). Detailed mechanistic studies in vitro demonstrated that dasatinib induced apoptosis of rat primary hepatocytes, which was marked by chromatin condensation, caspase-3 cleavage, and poly(ADP-ribose) polymerase (PARP) cleavage. Dasatinib was shown to increase intracellular reactive oxygen species (ROS), as measured by cellular oxidation of the fluorescent probe H_2_DCFDA (2′,7′-dichlorodihydrofluorescein diacetate), decrease GSH levels, and decrease superoxide dismutase (SOD) activity in a dose-dependent manner [58]. Dasatinib was also shown to activate Nrf2 and induce phosphorylation of p38 kinase, c-Jun N-terminal kinase (JNK), and extracellular signal-regulated kinase (ERK) [58]. Hepatic oxidative stress caused by dasatinib is proposed to involve the CYP3A4-mediated generation of electrophilic metabolites of dasatinib, including a quinoneimine, which can react with GSH and cysteine residues of proteins [15]. Pretreatment of rat hepatocytes with *N*-acetylcysteine attenuated the toxic effects of dasatinib [58].

### 3.2. Lapatinib

Lapatinib is a dual inhibitor of EGFR and HER2 tyrosine kinases, and lapatinib is approved in combination therapy for the treatment of advanced and metastatic breast cancer [59]. Lapatinib-induced hepatotoxicity is proposed to involve CYP3A-mediated *O*-dealkylation of lapatinib and further oxidation to form a reactive quinoneimine metabolite [18,60]. Hardy et al., reported studies on the role of metabolic activation on the cytotoxicity of lapatinib in HepaRG cells, a metabolically competent human hepatic cell line [61]. In this investigation, the *O*-dealkylated metabolite of lapatinib was significantly more cytotoxic (LD_50_ = 38.5 ± 1.1 µM) than lapatinib (LD_50_ = 84.9 ± 1.1 µM) itself (*p* < 0.01) [61]. GSH conjugation of electrophilic metabolites is an important detoxification pathway in vivo. GSH conjugates can undergo further metabolism in hepatic cell cultures and in vivo via the mercapturic acid pathway to form cysteine and *N*-acetylcysteine conjugates. Cysteine conjugates of the putative lapatinib quinoneimine were detected in HepaRG cells incubated with lapatinib. Induction of CYP3A4 by prototypical inducers, rifampin and dexamethasone, increased formation of *O*-dealkylated lapatinib and reactive metabolite cysteine conjugates [61]. CYP3A4 induction also potentiated the cytotoxicity of lapatinib [61]. Further, depletion of GSH using l-buthionine sulfoximine (BSO) enhanced the cytotoxicity of lapatinib. These findings suggest that CYP3A-mediated metabolic activation plays a key role in lapatinib-induced hepatocellular injury [61].

Eno et al. examined the cellular effects of lapatinib vs its *O*-dealkylated metabolite in HepG2 cells [62]. Mitochondrial respiration, as measured by oxygen consumption rate, was monitored using a Seahorse XF24 analyzer. The results of this study demonstrated that *O*-dealkylated lapatinib caused mitochondrial dysfunction in a time- and concentration-dependent manner. Treatment of HepG2 cells with 30 μM *O*-dealkylated lapatinib for 24 h significantly reduced basal and maximal oxygen consumption rate compared to untreated cells [62]. The parent drug lapatinib did not significantly alter mitochondrial respiration in HepG2 cells at the concentration tested (10 μM lapatinib). This observation is consistent with the fact that HepG2 cells lack the cytochrome P450 drug metabolism activity required to convert lapatinib to the potentially toxic metabolite. However, in HepG2 cells transfected with CYP3A4, 10 μM lapatinib significantly reduced the mitochondrial respiration [62]. This finding suggests that mitochondrial dysfunction is mediated by metabolism of lapatinib to the *O*-dealkylated metabolite. Pretreatment of HepG2 cells with BSO to deplete intracellular GSH followed by incubation with 20 μM *O*-dealkylated lapatinib resulted in a marked reduction in oxygen consumption rate compared to cells treated with *O*-dealkylated lapatinib alone. This indicates that GSH depletion enhanced *O*-dealkylated lapatinib-induced mitochondrial dysfunction [62].

Eno et al. further examined the effect of *O*-dealkylated lapatinib on oxidant stress responses in HepG2 cells [62]. Treatment of HepG2 cells with 40 µM *O*-dealkylated lapatinib increased mRNA expression of mitochondrial SOD2 but did not alter cytoplasmic SOD1 mRNA expression. Nrf2 activation was measured using an ARE-reporter assay [62]. *O*-dealkylated lapatinib was shown to activate Nrf2 in a concentration-dependent manner, and treatment of HepG2 cells with 40 µM *O*-dealkylated lapatinib for 4 h induced expression of Nrf2 target genes, NQO-1 and HO-1. Lapatinib-induced liver injury was also evaluated in vivo in wild-type and Nrf2-knockout mice [62]. Treatment of wild-type and Nrf2-knockout mice with lapatinib (400 mg/kg per day for 5 days) resulted in an increase in ALT and AST. The elevated ALT and AST levels returned back to normal in wild-type mice at day 5 of lapatinib treatment; however, ALT and AST levels remained high in Nrf2-knockout mice, suggesting that the Nrf2 pathway was important in protecting mice from hepatic injury [62].

### 3.3. Regorafenib

Regorafenib is a multi-targeted tyrosine kinase inhibitor approved for the treatment of metastatic colorectal cancer and imatinib- and sunitinib-resistant gastrointestinal stromal tumors [63]. The product label for regorafenib carries a black-box warning for hepatotoxicity. Liver injury associated with regorafenib is considered idiosyncratic, and is characterized primarily as hepatocellular injury. Weng et al., examined the mechanisms of regorafenib hepatotoxicity in isolated rat mitochondria and in primary hepatocytes [64]. Regorafenib was shown to cause uncoupling of oxidative phosphorylation, disruption of mitochondrial membrane potential, ATP depletion, and hepatocyte necrosis at clinically relevant concentrations (2.5–15 µM) of regorafenib [64].

### 3.4. Screening Tyrosine Kinase Inhibitors for Mitochondrial Toxicity

Studies by Krahenbuhl and colleagues have focused on defining the mitochondrial mechanisms of hepatocellular toxicity induced by tyrosine kinase inhibitors in hepatic cell lines, including HepG2 and HepaRG cells [65,66]. Paech et al., examined the toxic effects of four tyrosine kinase inhibitors (imatinib, sunitinib, lapatinib, and erlotinib) in HepG2 cells, HepaRG cells, and isolated mouse liver mitochondria [65]. In HepG2 cells, cytotoxicity, as characterized by the release of adenylate kinase, was shown for sunitinib at 5–10 µM, and for lapatinib at 10–20 µM, after 24–48 h of incubation. Imatinib and sunitinib (starting at 5 µM) decreased ATP content after 24–48 h of incubation. In HepaRG cells, induction of CYP3A4 by rifampicin (20 µM) enhanced the cytotoxicity of imatinib, lapatinib, and sunitinib, suggesting the role of toxic metabolites in mediating hepatocellular toxicity. This finding is consistent with previous reports by Hardy et al., and Teo et al., which showed that CYP3A induction potentiates the toxicity of lapatinib in hepatic cell cultures [61,67].

Imatinib and sunitinib demonstrated hepatotoxicity by increasing the production of reactive oxygen species, disrupting the mitochondrial membrane potential, depleting GSH content, and inducing apoptosis [65]. Lapatinib also decreased the mitochondrial membrane potential in mouse liver mitochondria and increased production of reactive oxygen species. As noted above, a limitation to this study was that most of the experiments were performed using HepG2 cells, which lack cytochrome P450 drug metabolism activity. However, the findings provide mechanistic insight into effects of the parent drugs on mitochondrial dysfunction. Among the tyrosine kinase inhibitors tested in this study, sunitinib displayed the most potent effects on cellular toxicity [65]. Sunitinib (20 µM) impaired glycolysis and beta oxidation in HepG2 cells, and sunitinib disrupted mitochondrial membrane potential starting at 1 µM in HepG2 cells and 10 µM in isolated mice mitochondria [65]. Sunitinib also diminished the respiration rate in HepG2 cells as concentrations increased by impairing complex I, which stimulated superoxide production. The accumulation of reactive oxygen species started at concentrations as low as 2 µM sunitinib and displayed a sharp increase at 20 µM sunitinib, which corresponded with a dramatic decrease in GSH content [65]. Consequently, the expression of superoxide dismutase 1 in the cytoplasm increased, which was likely due to an antioxidant defense response. The induction of apoptosis indicated by caspase 3/7 activity increased at 20 µM sunitinib, while PARP degradation occurred at 10 µM sunitinib. Although plasma concentrations of sunitinib are 0.1–0.3 µM, concentrations in the liver might be 10-fold higher [68]. This suggests that dose and concentration may play a role in the hepatotoxicity of sunitinib.

Mingard et al., examined the toxic effects of six tyrosine kinase inhibitors (crizotinib, dasatinib, pazotinib, ponatinib, regorafenib, and sorafenib) in HepG2 cells [66]. Regorafenib and sorafenib were shown to cause direct mitochondrial toxicity, as evidenced by increased ATP depletion in HepG2 cells in the presence of galactose compared to glucose [66]. Regorafenib and sorafenib (10 µM) decreased mitochondrial membrane potential in HepG2 cells [66]. Studies were also conducted in HepaRG cells to evaluate the impact of CYP-mediated metabolism of tyrosine kinase inhibitors on mitochondrial toxicity. Unlike studies with lapatinib, sunitinib, and imatinib in HepaRG cells, pretreatment of HepaRG cells with the CYP3A4 inducer rifampicin (20 µM) did not enhance the toxic effects of crizotinib, dasatinib, pazotinib, ponatinib, regorafenib, and sorafenib on mitochondrial membrane integrity or ATP depletion. Rather, intracellular ATP content modestly increased with rifampicin in cells treated with crizotinib (20 µM), dasatinib (50 µM), regorafenib (5 µM), and sorafenib (5 µM) [66]. Ponatinib, regorafenib, and sorafenib were shown to inhibit the mitochondrial electron transport chain. Crizotinib and ponatinib (20 µM) increased superoxide accumulation, and dasatinib and ponatinb (20 µM) caused intracellular GSH depletion. Based on their findings, Mingard et al. suggested a mitochondrial mechanism of hepatotoxicity for regorafenib, sorafenib, and potentially ponatinib, and a non-mitochondrial mechanism of hepatotoxicity for crizotinib, dasatinib, and pazopanib [66].

Zhang et al., screened 31 FDA-approved kinase inhibitors for mitochondrial toxicity using isolated rat mitochondria as a model [69]. Among the kinase inhibitors screened, sorafenib, regorafenib, and pazopanib were found to cause mitochondrial toxicity at therapeutic plasma concentrations (*C*_max_). The finding that sorafenib and regorafenib caused mitochondrial toxicity is consistent with reports by Mingard et al. [66]. Additional kinase inhibitors showed mitochondrial toxicity at higher concentrations (100-fold *C*_max_). As noted above, for some drugs, drug concentrations may be >10-fold higher in liver compared to plasma; thus, it is relevant to consider drug concentrations in the target tissue in addition to plasma concentrations.

## 4. Risk Factors of Hepatotoxicity

An important question to address is: What makes one patient more susceptible to idiosyncratic hepatotoxicity from tyrosine kinase inhibitors compared another patient who does not experience this serious adverse reaction? This is an area of intense ongoing investigation. A better understanding of the genetic and environmental factors that influence tyrosine kinase inhibitor toxicity risk is needed to predict and prevent these adverse events. A number of studies have focused on identifying genetic and environmental risk factors of hepatotoxicity related to tyrosine kinase inhibitors and other classes of drugs. A combination of drug properties and host-specific factors have been proposed to contribute to the development of drug-induced liver injury [70]. Daily dose and body burden of reactive metabolites are identified as important drug-specific factors [24]. Despite knowledge of chemical structural alerts that can undergo bioactivation and the practice of screening for reactive metabolite formation in early stages of drug discovery, hepatotoxicity remains a challenge for recently developed tyrosine kinase inhibitors [13,71,72,73,74]. The proposed host risk factors include human leukocyte antigen (HLA) risk alleles, co-administered drugs, cellular antioxidant capacity, disease state, nutritional status, age, sex, and genetic variations in drug metabolizing enzymes and transporters [70].

The HLA risk allele HLA-DRB1*07:01 (and HLA-DQA1*02:01) is associated with severe lapatinib-induced hepatotoxicity [75,76,77,78,79]. This suggests that lapatinib-related liver injury involves activation of the adaptive immune system, which may be triggered by the chemically reactive quinoneimine metabolite of lapatinib. *HLA-B*:*57-01 was shown to be a risk factor for pazopanib-induced hepatotoxicity, suggesting that an immune-mediated mechanism may be involved [80].

In addition to HLA alleles, co-administered drugs may also impact the risk of hepatotoxicity related to lapatinib. Teo et al., demonstrated that the CYP3A4 inducer dexamethasone increased the risk of lapatinib-induced hepatotoxicity in breast cancer patients [67]. While the highly polymorphic CYP3A5 has been shown to contribute to bioactivation on lapatinib [29,81,82], to our knowledge, no studies have definitively shown a correlation between *CYP3A5* genotype and lapatinib-induced hepatotoxicity.

Identifying the sources of variability in drug exposure for small molecule kinase inhibitors remains a challenge. This is relevant because variations in drug exposure can impact drug efficacy and toxicity. One study reported that *CYP3A5* and *CYP2D6* genotype and phenotype (poor metabolizer vs non-poor metabolizer) may affect the risk of severe hepatotoxicity induced by gefitinib in Japanese patients with non-small cell lung cancer [83]. Teo et al. reported that the relative risk for AST elevations due to sunitinib in an Asian patient population was 2.5 times lower in patients who express *CYP3A5* (individuals homozygous or heterozygous for CYP3A5*1) compared to *CYP3A5* non-expressers (CYP3A5*3/*3) [84]. Moreover, the relative risk for increases in ALT, AST, and total bilirubin was 2–3 times higher in patients with the ATP binding cassette (ABC) transporter genotype *ABCB1 CC* compared to *ABCB1 CT/TT* [84]. Whether polymorphisms in drug metabolism and transport genes affect patient exposure to reactive metabolites of tyrosine kinase inhibitors in vivo is unknown, and warrants further investigation.

## 5. Summary

Several tyrosine kinase inhibitors have been shown to undergo cytochrome P450-mediated bioactivation to form chemically reactive metabolites. However, several questions remain to establish the mechanistic link between reactive metabolites and drug toxicity: Does the tyrosine kinase inhibitor cause direct cellular toxicity and/or immune-mediated toxicity? What are the cellular targets and downstream mechanisms involved? Recent findings in the literature suggest that different tyrosine kinase inhibitors have different mechanisms of hepatotoxicity. Thus, a drug- and pathway-specific approach is necessary to better understand the mechanisms of hepatotoxicity associated with individual tyrosine kinase inhibitors. Future studies are needed to elucidate the downstream biochemical, cellular signaling, and immunologic events that lead to tyrosine kinase inhibitor-induced hepatocellular injury. In addition, ongoing research is needed to identify patient-specific risk factors for tyrosine kinase inhibitor-induced liver injury.

## 6. Methods

Articles were selected using a search on the PubMed database (https://www.ncbi.nlm.nih.gov/pubmed/). The search comprised the following keywords: tyrosine kinase inhibitor, reactive intermediate, reactive metabolite, bioactivation. The search was performed on 21 April 2018. In the initial search, a total of 28 results were generated in PubMed for the specific search of “tyrosine kinase inhibitor and reactive metabolite”; 15 results were generated from the search of “tyrosine kinase inhibitor and reactive intermediate”; and 14 results were generated from the search of “tyrosine kinase inhibitor and bioactivation”. Several overlapping articles were identified from the individual searches. The article titles were reviewed for relevance to the topic, and for the search of “tyrosine kinase inhibitor and reactive metabolite”, 21 articles were excluded because they were not related to cytochrome P450-mediated bioactivation. Additional review articles relevant to the topic were included, and articles cross-referenced in the initial articles were included in the final selection.

## Figures and Tables

**Table 1:** Summary of Kinase Inhibitors Approved for Clinical Use.

Drug	FDA Approval Year *^c^*	Target(s)	Indication	Primary Metabolic Pathway [11,12]
Imatinib	2001	Bcr-Abl, PDGFR, c-KIT	Ph^+^ CML, ALL, GIST, CEL	CYP3A4, CYP3A5
Gefitinib	2003	EGFR	NSCLC	CYP3A4, CYP3A5, CYP2D6, (CYP1A1 minor)
Erlotinib	2004	EGFR	NSCLC	CYP3A4, CYP1A2, CYP1A1
Sorafenib	2005	C-RAF, B-RAF, c-KIT, FLT3, VEGFR, PDGFR	HCC, RCC	CYP3A4, UGT1A9
Sunitinib *^a^*	2006	PDGFR, VEGFR, c-KIT, RET, CSF-1R, FLT3	RCC, GIST, pNET	CYP3A4 (major), CYP3A5, CYP1A1, CYP1A2
Dasatinib	2006	Bcr-Abl, SCR-family kinases, PDGFR, c-KIT, ephrin (EPH) receptor kinases	CML, ALL	CYP3A4 (major), FMO-3, UGT
Lapatinib *^a^*	2007	EGFR, HER-2	HER2+ breast cancer	CYP3A4, CYP3A5
Nilotinib	2007	Bcr-Abl	CML	CYP3A4, CYP2C8
Pazopanib *^a^*	2009	VEGFR, PDGFR, c-KIT	RCC	CYP3A4, (CYP1A2, CYP2C8 minor)
Vandetinib	2011	EGFR, VEGFR	MTC	CYP3A4, FMO-1 & -3
Crizotinib	2011	ALK	NSCLC	CYP3A4, CYP3A5
Vemurafenib	2011	BRAF	melanoma	CYP3A4
Ruxolitinib	2011	JAK	CIM, polycythemia vera, myelofibrosis	CYP3A4, CYP2C9
Ponatinib *^a^*	2012	ABL, KIT, RET	CML, ALL	CYP3A4, (CYP2C8, 2D6, 3A5 minor)
Regorafenib *^a^*	2012	RET, VEGFR, PDGFR, other	CRC, GIST	CYP3A4, UGT1A9
Axitinib	2012	VEGFR	RCC	CYP3A4, CYP3A5
Bosutinib	2012	Bcr-Abl	CML	CYP3A4
Cabozantinib	2012	MET, VEGFR	MTC	CYP3A4
Tofacitinib	2012	JAK	rheumatoid arthritis	CYP3A4, (CYP2C19 minor)
Ibrutinib	2013	BTK	mantle cell lymphoma, CLL	CYP3A, (CYP2D6 minor)
Afatinib *^b^*	2013	EGFR, HER2, HER4	NSCLC	negligible
Trametinib	2013	MEK	melanoma	carboxylesterases
Dabrafenib	2013	BRAF	melanoma	CYP2C8, CYP3A4
Ceritinib	2014	ALK	NSCLC	CYP3A
Nintedanib	2014	FGFR, PDGFR, VEGFR	pulmonary fibrosis	UGT1A1,7,8, (CYP3A4 minor)
Idelalisib *^a^*	2014	PI3K, B-cell R	CLL	CYP3A, AO, UGT1A4
Osimertinib *^b^*	2015	EGFR	NSCLC	CYP3A
Alectinib	2015	ALK	NSCLC	CYP3A4
Cobimetinib	2015	BRAF	advanced melanoma	CYP3A, UGT2B7
Lenvatinib	2015	VEGFR, PDGFR	thyroid cancer, RCC	CYP3A, AO
Palbociclib	2015	Cyclin-dependent kinase	HER2-, HR+ breast cancer	CYP3A, SULT2A1
Brigatinib	2017	ALK	NSCLC	CYP2C8, CYP3A4
Neratinib *^b^*	2017	EGFR, HER2, HER4	HER2+ breast cancer	CYP3A4, FMO
Ribociclib	2017	Cyclin-dependent kinase	EGFR-, HR+ breast cancer	CYP3A4

*^a^* Kinase inhibitors whose product labels have a black-box warning for hepatotoxicity; *^b^* Kinase inhibitors in which covalent modification is involved in the pharmacologic mechanism of action; *^c^* Drug prescribing information was used to confirm FDA approval year, target(s), indication, and metabolic pathway(s).

**Table 2:** Tyrosine Kinase Inhibitors Reported to Form Reactive Metabolites.

Drug	Daily Dose *^a^*	Bioactivation Pathway	Reactive Intermediate(s)	Reference
Dasatinib	100–140 mg	CYP3A4	Quinoneimine, Imine methide	Li et al., 2009 [15].
Gefitinib	250 mg	CYP3A4 CYP1A1	Quinoneimine	Li et al., 2009 [16].
Erlotinib	150 mg	CYP3A4, CYP1A1 CYP3A4/5	Quinoneimine, Ketene intermediate	Li et al., 2010 [17]. Zhao et al., 2018 [31].
Lapatinib	1250–1500 mg	CYP3A4/5	Quinoneimine, Nitroso intermediate	Teng et al., 2010 [18]. Takakusa et al., 2011 [19].
Imatinib	300–800 mg	CYP3A4	Imine, Imine methide	Li et al., 2014 [20].
Axitinib	5 mg BID	CYP3A4	Possible epoxide	Wang et al., 2014 [21].
Ponatinib	45 mg	CYP1A1	Possible epoxide	Lin et al., 2017 [22].
Sunitinib	37.5–50 mg	CYP1A2, CYP3A4	Quinoneimine	Amaya et al., 2018 [23].
Saracatinib (azd0530)		CYP3A4	*ortho*-Quinone	Chen et al., 2016 [33].
Masitinib			Imine, Imine carbonyl	Amer et al., 2017 [34].

*^a^* Dosage information was obtained from drug prescribing information.

## Abbreviations

ALK	anaplastic lymphoma kinase
ALL	acute lymphatic leukemia
ALT	alanine aminotransferase
AO	aldehyde oxidase
AST	aspartate aminotransferase
AUC	area under the curve
BID	twice daily
BSO	buthionine sulfoximine
BTK	Bruton’s tyrosine kinase
CEL	chronic eosinophilic leukemia
CIM	chronic idiopathic myelofibrosis
CLL	chronic lymphocytic leukemia
CML	chronic myeloid leukemia
CRC	colorectal cancer
CYP	cytochrome P450
EGFR	epidermal growth factor receptor
FGFR	fibroblast growth factor receptor
GIST	gastrointestinal stromal tumors
HCC	hepatocellular carcinoma
HER2	human epidermal growth factor receptor 2
HR	hormone receptor
JAK	Janus kinase
MEK	mitogen-activated extracellular signal-regulated kinase
MTC	metastatic thyroid cancer
Nrf2	nuclear factor erythroid 2-related factor
NSCLC	non-small cell lung cancer
PDGFR	platelet-derived growth factor receptor
pNET	pancreatic neuroendocrine tumors
RCC	renal cell carcinoma
VEGFR	vascular endothelial growth factor receptor

## References

[1] Cohen P. (2002). The origins of protein phosphorylation. Nat. Cell Biol..

[2] Krause D.S., Van Etten R.A. (2005). Tyrosine kinases as targets for cancer therapy. N. Engl. J. Med..

[3] Cohen M.H., Williams G., Johnson J.R., Duan J., Gobburu J., Rahman A., Benson K., Leighton J., Kim S.K., Wood R. (2002). Approval Summary for Imatinib Mesylate Capsules in the Treatment of Chronic Myelogenous Leukemia. Clin. Cancer Res..

[4] Wu P., Nielsen T.E., Clausen M.H. (2016). Small-molecule kinase inhibitors: An analysis of FDA-approved drugs. Drug Discov. Today.

[5] Liu S., Kurzrock R. (2014). Toxicity of targeted therapy: Implications for response and impact of genetic polymorphisms. Cancer Treat. Rev..

[6] Shah R., Morganroth J., Shah D. (2013). Hepatotoxicity of Tyrosine Kinase Inhibitors: Clinical and Regulatory Perspectives. Drug Saf..

[7] Teo Y.L., Ho H.K., Chan A. (2013). Risk of tyrosine kinase inhibitors-induced hepatotoxicity in cancer patients: A meta-analysis. Cancer Treat. Rev..

[8] Karczmarek-Borowska B., Salek-Zan A. (2015). Hepatotoxicity of molecular targeted therapy. Contemp. Oncol..

[9] Ghatalia P., Je Y., Mouallem N.E., Nguyen P.L., Trinh Q.D., Sonpavde G., Choueiri T.K. (2015). Hepatotoxicity with vascular endothelial growth factor receptor tyrosine kinase inhibitors: A meta-analysis of randomized clinical trials. Crit. Rev. Oncol. Hematol..

[10] Russmann S., Kullak-Ublick G.A., Grattagliano I. (2009). Current concepts of mechanisms in drug-induced hepatotoxicity. Curr. Med. Chem..

[11] Duckett D.R., Cameron M.D. (2010). Metabolism considerations for kinase inhibitors in cancer treatment. Expert Opin. Drug Metab. Toxicol..

[12] Teo Y.L., Ho H.K., Chan A. (2015). Metabolism-related pharmacokinetic drug-drug interactions with tyrosine kinase inhibitors: Current understanding, challenges and recommendations. Br. J. Clin. Pharmacol..

[13] Stepan A.F., Walker D.P., Bauman J., Price D.A., Baillie T.A., Kalgutkar A.S., Aleo M.D. (2011). Structural alert/reactive metabolite concept as applied in medicinal chemistry to mitigate the risk of idiosyncratic drug toxicity: A perspective based on the critical examination of trends in the top 200 drugs marketed in the United States. Chem. Res. Toxicol..

[14] Teo Y.L., Ho H.K., Chan A. (2015). Formation of reactive metabolites and management of tyrosine kinase inhibitor-induced hepatotoxicity: A literature review. Expert Opin. Drug Metab. Toxicol..

[15] Li X., He Y., Ruiz C.H., Koenig M., Cameron M.D., Vojkovsky T. (2009). Characterization of dasatinib and its structural analogs as CYP3A4 mechanism-based inactivators and the proposed bioactivation pathways. Drug Metab. Dispos..

[16] Li X., Kamenecka T.M., Cameron M.D. (2009). Bioactivation of the epidermal growth factor receptor inhibitor gefitinib: Implications for pulmonary and hepatic toxicities. Chem. Res. Toxicol..

[17] Li X., Kamenecka T.M., Cameron M.D. (2010). Cytochrome P450-mediated bioactivation of the epidermal growth factor receptor inhibitor erlotinib to a reactive electrophile. Drug Metab. Dispos..

[18] Teng W.C., Oh J.W., New L.S., Wahlin M.D., Nelson S.D., Ho H.K., Chan E.C.Y. (2010). Mechanism-Based Inactivation of Cytochrome P450 3A4 by Lapatinib. Mol. Pharmacol..

[19] Takakusa H., Wahlin M.D., Zhao C., Hanson K.L., New L.S., Chan E.C.Y., Nelson S.D. (2011). Metabolic Intermediate Complex Formation of Human Cytochrome P450 3A4 by Lapatinib. Drug Metab. Dispos..

[20] Li A.C., Yu E., Ring S.C., Chovan J.P. (2014). Structural identification of imatinib cyanide adducts by mass spectrometry and elucidation of bioactivation pathway. Rapid Commun. Mass Spectrom..

[21] Wang Y., Wang M., Qi H., Pan P., Hou T., Li J., He G., Zhang H. (2014). Pathway-dependent inhibition of paclitaxel hydroxylation by kinase inhibitors and assessment of drug-drug interaction potentials. Drug Metab. Dispos..

[22] Lin D., Kostov R., Huang J.T., Henderson C.J., Wolf C.R. (2017). Novel Pathways of Ponatinib Disposition Catalyzed By CYP1A1 Involving Generation of Potentially Toxic Metabolites. J. Pharmacol. Exp. Ther..

[23] Amaya G.M., Durandis R., Bourgeois D.S., Perkins J.A., Abouda A.A., Wines K.J., Mohamud M., Starks S.A., Daniels R.N., Jackson K.D. (2018). Cytochromes P450 1A2 and 3A4 Catalyze the Metabolic Activation of Sunitinib. Chem. Res. Toxicol..

[24] Obach R.S., Kalgutkar A.S., Soglia J.R., Zhao S.X. (2008). Can in vitro metabolism-dependent covalent binding data in liver microsomes distinguish hepatotoxic from nonhepatotoxic drugs? An analysis of 18 drugs with consideration of intrinsic clearance and daily dose. Chem. Res. Toxicol..

[25] Bauman J.N., Kelly J.M., Tripathy S., Zhao S.X., Lam W.W., Kalgutkar A.S., Obach R.S. (2009). Can in vitro metabolism-dependent covalent binding data distinguish hepatotoxic from nonhepatotoxic drugs? An analysis using human hepatocytes and liver S-9 fraction. Chem. Res. Toxicol..

[26] Kenny J.R., Mukadam S., Zhang C., Tay S., Collins C., Galetin A., Khojasteh S.C. (2012). Drug-drug interaction potential of marketed oncology drugs: In vitro assessment of time-dependent cytochrome P450 inhibition, reactive metabolite formation and drug-drug interaction prediction. Pharm. Res..

[27] Evans D.C., Watt A.P., Nicoll-Griffith D.A., Baillie T.A. (2004). Drug-protein adducts: An industry perspective on minimizing the potential for drug bioactivation in drug discovery and development. Chem. Res. Toxicol..

[28] Van Erp N.P., Gelderblom H., Guchelaar H.-J. (2009). Clinical pharmacokinetics of tyrosine kinase inhibitors. Cancer Treat. Rev..

[29] Chan E.C.Y., New L.S., Chua T.B., Yap C.W., Ho H.K., Nelson S.D. (2012). Interaction of Lapatinib with Cytochrome P450 3A5. Drug Metab. Dispos..

[30] Filppula A.M., Neuvonen P.J., Backman J.T. (2014). In vitro assessment of time-dependent inhibitory effects on CYP2C8 and CYP3A activity by fourteen protein kinase inhibitors. Drug Metab. Dispos..

[31] Zhao H., Li S., Yang Z., Peng Y., Chen X., Zheng J. (2018). Identification of Ketene-Reactive Intermediate of Erlotinib Possibly Responsible for Inactivation of P450 Enzymes. Drug Metab. Dispos..

[32] Hu-Lowe D.D., Zou H.Y., Grazzini M.L., Hallin M.E., Wickman G.R., Amundson K., Chen J.H., Rewolinski D.A., Yamazaki S., Wu E.Y. (2008). Nonclinical Antiangiogenesis and Antitumor Activities of Axitinib (AG-013736), an Oral, Potent, and Selective Inhibitor of Vascular Endothelial Growth Factor Receptor Tyrosine Kinases 1, 2, 3. Clin. Cancer Res..

[33] Chen J., Peng Y., Zheng J. (2016). Cytochrome P450 Mediated Bioactivation of Saracatinib. Chem. Res. Toxicol..

[34] Amer S.M., Kadi A.A., Darwish H.W., Attwa M.W. (2017). Identification and characterization of in vitro phase I and reactive metabolites of masitinib using a LC-MS/MS method: Bioactivation pathway elucidation. RSC Adv..

[35] Lipton J.H., Chuah C., Guerci-Bresler A., Rosti G., Simpson D., Assouline S., Etienne G., Nicolini F.E., le Coutre P., Clark R.E. (2016). Ponatinib versus imatinib for newly diagnosed chronic myeloid leukaemia: An international, randomised, open-label, phase 3 trial. Lancet Oncol..

[36] Guengerich F.P., de Montellano P.R.O. (2015). Human Cytochrome P450 Enzymes. Cytochrome P450: Structure, Mechanism, and Biochemistry.

[37] Chow L.Q., Eckhardt S.G. (2007). Sunitinib: From rational design to clinical efficacy. J. Clin. Oncol..

[38] Goodman V.L., Rock E.P., Dagher R., Ramchandani R.P., Abraham S., Gobburu J.V., Booth B.P., Verbois S.L., Morse D.E., Liang C.Y. (2007). Approval summary: Sunitinib for the treatment of imatinib refractory or intolerant gastrointestinal stromal tumors and advanced renal cell carcinoma. Clin. Cancer Res..

[39] Xie C., Zhou J., Guo Z., Diao X., Gao Z., Zhong D., Jiang H., Zhang L., Chen X. (2013). Metabolism and bioactivation of famitinib, a novel inhibitor of receptor tyrosine kinase, in cancer patients. Br. J. Pharmacol..

[40] Green T.P., Fennell M., Whittaker R., Curwen J., Jacobs V., Allen J., Logie A., Hargreaves J., Hickinson D.M., Wilkinson R.W. (2009). Preclinical anticancer activity of the potent, oral Src inhibitor AZD0530. Mol. Oncol..

[41] Attwa M.W., Kadi A.A., Darwish H.W., Alrabiah H. (2018). LC-MS/MS reveals the formation of reactive ortho-quinone and iminium intermediates in saracatinib metabolism: Phase I metabolic profiling. Clin. Chim. Acta.

[42] Dubreuil P., Letard S., Ciufolini M., Gros L., Humbert M., Casteran N., Borge L., Hajem B., Lermet A., Sippl W. (2009). Masitinib (AB1010), a potent and selective tyrosine kinase inhibitor targeting KIT. PLoS ONE.

[43] Committee for Medicinal Products for Human Use (CHMP) (2014). Masitinib (Masiviera). Assessment Report.

[44] Li A.P. (2002). A review of the common properties of drugs with idiosyncratic hepatotoxicity and the “multiple determinant hypothesis” for the manifestation of idiosyncratic drug toxicity. Chem. Biol. Interact..

[45] Mosedale M., Watkins P.B. (2017). Drug-induced liver injury: Advances in mechanistic understanding that will inform risk management. Clin. Pharmacol. Ther..

[46] Tailor A., Faulkner L., Naisbitt D.J., Park B.K. (2015). The chemical, genetic and immunological basis of idiosyncratic drug-induced liver injury. Hum. Exp. Toxicol..

[47] Park K.B., Kitteringham N.R., Maggs J.L., Pirmohamed M., Williams D.P. (2005). The role of metabolic activation in drug-induced hepatotoxicity. Annu. Rev. Pharmacol. Toxicol..

[48] Pauli-Magnus C., Meier P.J. (2006). Hepatobiliary transporters and drug-induced cholestasis. Hepatology.

[49] Feng B., Xu J.J., Bi Y.A., Mireles R., Davidson R., Duignan D.B., Campbell S., Kostrubsky V.E., Dunn M.C., Smith A.R. (2009). Role of hepatic transporters in the disposition and hepatotoxicity of a HER2 tyrosine kinase inhibitor CP-724,714. Toxicol. Sci..

[50] McGill M.R., Yan H.M., Ramachandran A., Murray G.J., Rollins D.E., Jaeschke H. (2011). HepaRG cells: A human model to study mechanisms of acetaminophen hepatotoxicity. Hepatology.

[51] Aninat C., Piton A., Glaise D., Le Charpentier T., Langouet S., Morel F., Guguen-Guillouzo C., Guillouzo A. (2006). Expression of cytochromes P450, conjugating enzymes and nuclear receptors in human hepatoma HepaRG cells. Drug Metab. Dispos..

[52] Guillouzo A., Corlu A., Aninat C., Glaise D., Morel F., Guguen-Guillouzo C. (2007). The human hepatoma HepaRG cells: A highly differentiated model for studies of liver metabolism and toxicity of xenobiotics. Chem. Biol. Interact..

[53] Andersson T.B., Kanebratt K.P., Kenna J.G. (2012). The HepaRG cell line: A unique in vitro tool for understanding drug metabolism and toxicology in human. Expert Opin. Drug Metab. Toxicol..

[54] Antherieu S., Chesne C., Li R., Camus S., Lahoz A., Picazo L., Turpeinen M., Tolonen A., Uusitalo J., Guguen-Guillouzo C. (2010). Stable expression, activity, and inducibility of cytochromes P450 in differentiated HepaRG cells. Drug Metab. Dispos..

[55] Marroquin L.D., Hynes J., Dykens J.A., Jamieson J.D., Will Y. (2007). Circumventing the Crabtree effect: Replacing media glucose with galactose increases susceptibility of HepG2 cells to mitochondrial toxicants. Toxicol. Sci..

[56] Itoh K., Wakabayashi N., Katoh Y., Ishii T., Igarashi K., Engel J.D., Yamamoto M. (1999). Keap1 represses nuclear activation of antioxidant responsive elements by Nrf2 through binding to the amino-terminal Neh2 domain. Genes Dev..

[57] Dinkova-Kostova A.T., Holtzclaw W.D., Cole R.N., Itoh K., Wakabayashi N., Katoh Y., Yamamoto M., Talalay P. (2002). Direct evidence that sulfhydryl groups of Keap1 are the sensors regulating induction of phase 2 enzymes that protect against carcinogens and oxidants. Proc. Natl. Acad. Sci. USA.

[58] Xue T., Luo P., Zhu H., Zhao Y., Wu H., Gai R., Wu Y., Yang B., Yang X., He Q. (2012). Oxidative stress is involved in Dasatinib-induced apoptosis in rat primary hepatocytes. Toxicol. Appl. Pharmacol..

[59] Medina P.J., Goodin S. (2008). Lapatinib: A dual inhibitor of human epidermal growth factor receptor tyrosine kinases. Clin. Ther..

[60] Castellino S., O’Mara M., Koch K., Borts D.J., Bowers G.D., MacLauchlin C. (2012). Human Metabolism of Lapatinib, a Dual Kinase Inhibitor: Implications for Hepatotoxicity. Drug Metab. Dispos..

[61] Hardy K.D., Wahlin M.D., Papageorgiou I., Unadkat J.D., Rettie A.E., Nelson S.D. (2014). Studies on the Role of Metabolic Activation in Tyrosine Kinase Inhibitor–Dependent Hepatotoxicity: Induction of CYP3A4 Enhances the Cytotoxicity of Lapatinib in HepaRG Cells. Drug Metab. Dispos..

[62] Eno M.R., El-Gendy Bel D., Cameron M.D. (2016). P450 3A-Catalyzed O-Dealkylation of Lapatinib Induces Mitochondrial Stress and Activates Nrf2. Chem. Res. Toxicol..

[63] Crona D.J., Keisler M.D., Walko C.M. (2013). Regorafenib: A novel multitargeted tyrosine kinase inhibitor for colorectal cancer and gastrointestinal stromal tumors. Ann. Pharmacother..

[64] Weng Z., Luo Y., Yang X., Greenhaw J.J., Li H., Xie L., Mattes W.B., Shi Q. (2015). Regorafenib impairs mitochondrial functions, activates AMP-activated protein kinase, induces autophagy, and causes rat hepatocyte necrosis. Toxicology.

[65] Paech F., Bouitbir J., Krahenbuhl S. (2017). Hepatocellular Toxicity Associated with Tyrosine Kinase Inhibitors: Mitochondrial Damage and Inhibition of Glycolysis. Front. Pharmacol..

[66] Mingard C., Paech F., Bouitbir J., Krahenbuhl S. (2018). Mechanisms of toxicity associated with six tyrosine kinase inhibitors in human hepatocyte cell lines. J. Appl. Toxicol..

[67] Teo Y.L., Saetaew M., Chanthawong S., Yap Y.S., Chan E.C., Ho H.K., Chan A. (2012). Effect of CYP3A4 inducer dexamethasone on hepatotoxicity of lapatinib: Clinical and in vitro evidence. Breast Cancer Res. Treat..

[68] Lau C.L., Chan S.T., Selvaratanam M., Khoo H.W., Lim A.Y., Modamio P., Marino E.L., Segarra I. (2015). Sunitinib-ibuprofen drug interaction affects the pharmacokinetics and tissue distribution of sunitinib to brain, liver, and kidney in male and female mice differently. Fundam. Clin. Pharmacol..

[69] Zhang J., Salminen A., Yang X., Luo Y., Wu Q., White M., Greenhaw J., Ren L., Bryant M., Salminen W. (2017). Effects of 31 FDA approved small-molecule kinase inhibitors on isolated rat liver mitochondria. Arch. Toxicol..

[70] Chen M., Suzuki A., Borlak J., Andrade R.J., Lucena M.I. (2015). Drug-induced liver injury: Interactions between drug properties and host factors. J. Hepatol..

[71] Walsh J.S., Miwa G.T. (2011). Bioactivation of drugs: Risk and drug design. Annu. Rev. Pharmacol. Toxicol..

[72] Stachulski A.V., Baillie T.A., Park B.K., Obach R.S., Dalvie D.K., Williams D.P., Srivastava A., Regan S.L., Antoine D.J., Goldring C.E. (2013). The generation, detection, and effects of reactive drug metabolites. Med. Res. Rev..

[73] Kalgutkar A.S., Dalvie D. (2015). Predicting toxicities of reactive metabolite-positive drug candidates. Annu. Rev. Pharmacol. Toxicol..

[74] Gomez-Lechon M.J., Tolosa L., Donato M.T. (2016). Metabolic activation and drug-induced liver injury: In vitro approaches for the safety risk assessment of new drugs. J. Appl. Toxicol..

[75] Spraggs C.F., Budde L.R., Briley L.P., Bing N., Cox C.J., King K.S., Whittaker J.C., Mooser V.E., Preston A.J., Stein S.H. (2011). HLA-DQA1*02:01 Is a Major Risk Factor for Lapatinib-Induced Hepatotoxicity in Women With Advanced Breast Cancer. J. Clin. Oncol..

[76] Spraggs C.F., Parham L.R., Hunt C.M., Dollery C.T. (2012). Lapatinib-Induced Liver Injury Characterized by Class II HLA and Gilbert’s Syndrome Genotypes. Clin. Pharmacol. Ther..

[77] Spraggs C.F., Xu C.-F., Hunt C.M. (2013). Genetic characterization to improve interpretation and clinical management of hepatotoxicity caused by tyrosine kinase inhibitors. Pharmacogenomics.

[78] Schaid D.J., Spraggs C.F., McDonnell S.K., Parham L.R., Cox C.J., Ejlertsen B., Finkelstein D.M., Rappold E., Curran J., Cardon L.R. (2014). Prospective validation of HLA-DRB1*07:01 allele carriage as a predictive risk factor for lapatinib-induced liver injury. J. Clin. Oncol..

[79] Parham L.R., Briley L.P., Li L., Shen J., Newcombe P.J., King K.S., Slater A.J., Dilthey A., Iqbal Z., McVean G. (2016). Comprehensive genome-wide evaluation of lapatinib-induced liver injury yields a single genetic signal centered on known risk allele HLA-DRB1[ast]07:01. Pharmacogenomics J..

[80] Xu C.F., Johnson T., Wang X., Carpenter C., Graves A.P., Warren L., Xue Z., King K.S., Fraser D.J., Stinnett S. (2016). HLA-B*57:01 Confers Susceptibility to Pazopanib-Associated Liver Injury in Patients with Cancer. Clin. Cancer Res..

[81] Ho H.K., Chan J.C., Hardy K.D., Chan E.C. (2015). Mechanism-based inactivation of CYP450 enzymes: A case study of lapatinib. Drug Metab. Rev..

[82] Towles J.K., Clark R.N., Wahlin M.D., Uttamsingh V., Rettie A.E., Jackson K.D. (2016). Cytochrome P450 3A4 and CYP3A5-Catalyzed Bioactivation of Lapatinib. Drug Metab. Dispos..

[83] Sugiyama E., Umemura S., Nomura S., Kirita K., Matsumoto S., Yoh K., Niho S., Ohmatsu H., Tsuboi M., Ohe Y. (2015). Impact of single nucleotide polymorphisms on severe hepatotoxicity induced by EGFR tyrosine kinase inhibitors in patients with non-small cell lung cancer harboring EGFR mutations. Lung Cancer.

[84] Teo Y.L., Wee H.L., Chue X.P., Chau N.M., Tan M.H., Kanesvaran R., Wee H.L., Ho H.K., Chan A. (2016). Effect of the CYP3A5 and ABCB1 genotype on exposure, clinical response and manifestation of toxicities from sunitinib in Asian patients. Pharmacogenomics J..

